# Computed tomographic appearance of laryngeal lesions in 7 dogs

**DOI:** 10.3389/fvets.2025.1633591

**Published:** 2025-09-04

**Authors:** Anna Slusarek, Annick Hamaide, Michaël Lefebvre, Marianne Heimann, Frédéric Billen, Géraldine Bolen

**Affiliations:** ^1^Department of Companion Animal Clinical Sciences, Faculty of Veterinary Medicine, University of Liège, Liège, Belgium; ^2^Centre vétérinaire de Spécialistes Caladrius, Wavre, Belgium; ^3^Anapet srl, Synlab, Heppignies, Belgium

**Keywords:** computed tomography, larynx, canine, mass, tumor, dog

## Abstract

**Objective:**

To describe the computed tomographic (CT) features of neoplastic and inflammatory laryngeal masses. The authors hypothesized that specific CT features may help differentiate between these two origins and that regional lymph nodes would be larger in cases of laryngeal neoplasia.

**Methods:**

Medical records from two veterinary referral hospitals were screened for dogs diagnosed with either an inflammatory or neoplastic laryngeal mass who underwent CT scans of the neck. Information retrieved from medical records included signalment, physical examination findings, CT scan findings, and definitive diagnosis of the laryngeal mass based on cytological or histopathological results.

**Results:**

Four dogs had laboratory reports compatible with a malignant neoplasia and three with an inflammatory process. The shape of the mass was defined as “ovoid” in all neoplastic masses and as “thickening” in cases of inflammatory processes. Masses were of various sizes (median length: 42 mm, range: 26–82 mm) and either unilateral (1/4 and 2/3 of neoplastic and inflammatory masses respectively) or bilateral. They were described as mineralized (1/4 and 1/3) and as having either an internal (1/4), external (2/4) growth pattern or both (1/4, 3/3). All masses had ill-defined margins and showed heterogeneous contrast enhancement. Two neoplastic and two inflammatory masses had a cavitary aspect. All but one case were associated with regional lymphadenopathy. Thyroid cartilage destruction was observed with two neoplastic and two inflammatory masses.

**Clinical relevance:**

This case series describes CT features of laryngeal masses. The shape of the laryngeal mass may assist in determining its nature, inflammatory process was defined as “thickening” of the larynx and neoplasia as “ovoid”-shaped, whereas other studied features were inconsistently observed in both neoplastic and inflammatory conditions.

## Introduction

Neoplasms of the larynx are rare in dogs (21). Different types of laryngeal tumors have been reported in the dog, of which squamous cell carcinoma is the most common (12, 21). Adenocarcinoma, fibrosarcoma, osteosarcoma, extramedullary plasmocytoma, chondrosarcoma, rhabdomyosarcoma, lymphoma, mast cell tumor, granular cell tumors and melanoma are also described (16, 21). Benign tumors of the larynx include rhabdomyoma, leiomyoma, lipoma, osteochondroma and oncocytoma which is a benign tumor unique to the larynx (12, 14). Clinical signs typically include progressive dysphonia, stridor, stertor, cough, exercise intolerance, dysphagia, inappetence and dyspnea (11, 12, 21).

The most common diagnostic technique is direct visualization during laryngoscopy (12, 21). Regional radiographs can also reveal a space occupying soft tissue mass and additional thoracic radiographs can be performed in order to screen for pulmonary metastases.

These diagnostic tools do not permit characterization of the total volume and the margins of the laryngeal mass. Computed tomography (CT) is increasingly being applied for investigation and surgical planning in small animal patients with suspected cranial neck masses of unknown origin (8).

Laryngeal neoplasms in small animals have been described on CT as asymmetric intramural masses with intraluminal and possible extraluminal extension, with heterogeneous soft tissue attenuation and moderate contrast enhancement. Other features include a hypoattenuating necrotic or hemorrhagic center and lysis of the hyoid bones (19).

Since these criteria have been published, only few studies describe the CT features of neoplastic and non-neoplastic laryngeal masses including two case reports with CT features of one laryngeal rhabdomyoma and one laryngeal osseous metaplasia and two studies describing the CT features of neck masses involving the larynx but in which the larynx was not the primary origin of the mass (3, 6, 15, 20). More recently, Dixon et al. (5) provided a CT description of 11 confirmed laryngeal lesions, inflammatory and neoplastic, but without comparing them to each other and Matz et al. (11) reported 4 laryngeal tumors without exhaustive description of the CT features (5, 11).

To the authors’ knowledge, there is limited literature on the topic, especially regarding the description of inflammatory lesions, and no published description of the CT features that discriminate between neoplastic and inflammatory laryngeal masses.

CT could be used more effectively to help radiologists prioritize the differential diagnosis, assess the potential need for surgical management, and determine the size of the mass, which is not always feasible with endoscopy.

Therefore, the purpose of this study was to describe the CT features of laryngeal neoplastic and inflammatory masses in dogs. We hypothesized that specific CT features could help differentiate between these two etiologies, and that regional lymph nodes would be larger in cases of laryngeal neoplasia.

## Materials and methods

Dogs with laryngeal masses were enrolled in this multicenter, retrospective, case series study. Databases of medical records from two referral hospitals were screened from January 2013 to August 2024. Inclusion criteria included CT scan of the neck as well as biopsy or fine needle aspiration of the laryngeal mass reviewed by an European College of Veterinary Pathologists or an American College of Veterinary Pathologists diplomate. Exclusion criteria included patients with a mass effect involving the larynx, where the larynx was not the primary site of origin of the mass. Decisions for case inclusion were made by a European College of Veterinary Diagnostic Imaging -certified veterinary radiologist (G.B.) and a diagnostic imaging resident (A.S.). Breed, age, sex and main clinical and laboratory findings were recorded.

Dogs were included if they had a CT of the head and neck performed less than 2 weeks before the cytological or the histopathological diagnosis. Computed tomographic images were acquired with a 16-multislice CT scanner between 2013 and April 2019 (Siemens, Somatom 16) and a 64-multislice CT scanner between April 2019 and August 2024 (Siemens, Somatom Confidence 64) in one referral hospital and a 80-slice CT scanner (Aquilion Lightning SP 80-slice CT scanner, Canon Medical Systems) in second referral center. The acquisition method for all the cases included a scan slice thickness of 1 mm, 120 kV and variable mA (automatic exposure control).

The CT study had to be of diagnostic quality, acquired before and within 5 min after intravenous contrast medium injection (Ultravist 300 mgI/ml, 2 ml/kg, Iopromide, Bayer), prior to biopsy or fine needle aspiration. All CT examinations were performed under general anesthesia with an endotracheal tube in place.

All imaging studies were reviewed by a board-certified radiologist (European College of Veterinary Diagnostic Imaging, G.B.) and a diagnostic imaging resident (A.S.). Both reviewers were blinded concerning the final diagnosis of the mass while assessing the CT images. In cases where quantitative measurements differed between reviewers, the values were averaged. For dichotomous variables, disagreements were resolved through discussion until a consensus was reached.

All assessments and measurements were performed using dedicated DICOM viewer software (Horos, Horosproject.org, Annapolis, MD USA). A window width of 400 Houndsfield units (HU) and a window level of 40 HU were used. Thoracic CT images were reconstructed with a high frequency algorithm and were also reviewed when available.

The laryngeal appearance was documented with the following criteria: shape (thickening or ovoid-shaped), lateralization (unilateral or bilateral), symmetry, subjective homogeneity or heterogeneity before and after intravenous contrast medium injection, external or internal growth pattern or both, margins (ill-defined or well-defined), cavitation (present or absent), mineralization (present or absent) and invasion of adjacent structures. A lesion was classified as having a cavitary component when at least one focal hypoattenuating zone of this lesion showed no contrast enhancing after intravenous contrast medium administration. The classification into external or internal growth pattern was deliberately chosen, as the classification into intramural/extramural or concentric/eccentric did not seem appropriate in these cases.

The largest linear dimension of the mass-like lesion of the larynx was measured in three planes on postcontrast images.

The size and appearance of each regional lymph node were recorded, including medial retropharyngeal, mandibular, parotid, deep, and superficial cervical lymph nodes. A ratio of the short to long axis was calculated for each one of these lymph nodes and the lymph node was considered as enlarged if this ratio was > 0.5. The attenuation pattern (homogeneous or heterogeneous) was also assessed.

Regions of interest were drawn on each laryngeal lesion and each enlarged lymph node before and after intravenous contrast medium injection in order to determine if there was contrast enhancement or not.

When available, thoracic CT scan findings were recorded.

## Results

### Signalment and clinical findings

Seven dogs met the inclusion criteria. Age ranged from 2 to 12 years with a median age of 9 years. Mixed breed accounted for 3 of the 7 dogs and one each of other breeds (Border Collie, Beagle, English Springer Spaniel, White Swiss Shepherd). There were two spayed females, two intact males, and three neutered males. The median bodyweight was 31 kg (range: 17.8–35 kg).

Dysphonia was the most common clinical sign (5/7), followed by dyspnea (4/7). Dysphagia (2/7), exercise intolerance (2/7) and lethargy (2/7) were also reported. The duration of clinical signs was not consistently reported by the owners; however, for the three cases where this information was available, the duration ranged from 2 weeks to several months. On physical examination, a cervical mass was palpated in 3 out of the 7 cases.

Final diagnosis included neoplasia based on histology in 2 dogs (i.e., squamous cell carcinoma) and cytology in 2 dogs (i.e., carcinoma suspected of squamous cell origin), and inflammatory tissue based on histology in 2 dogs and cytology in 1 dog. Regarding the inflammatory tissues, all were characterized as being composed of lymphocytes, neutrophils and plasma cells, with no atypical cell present.

### Diagnostic imaging findings

**Figure 1:**
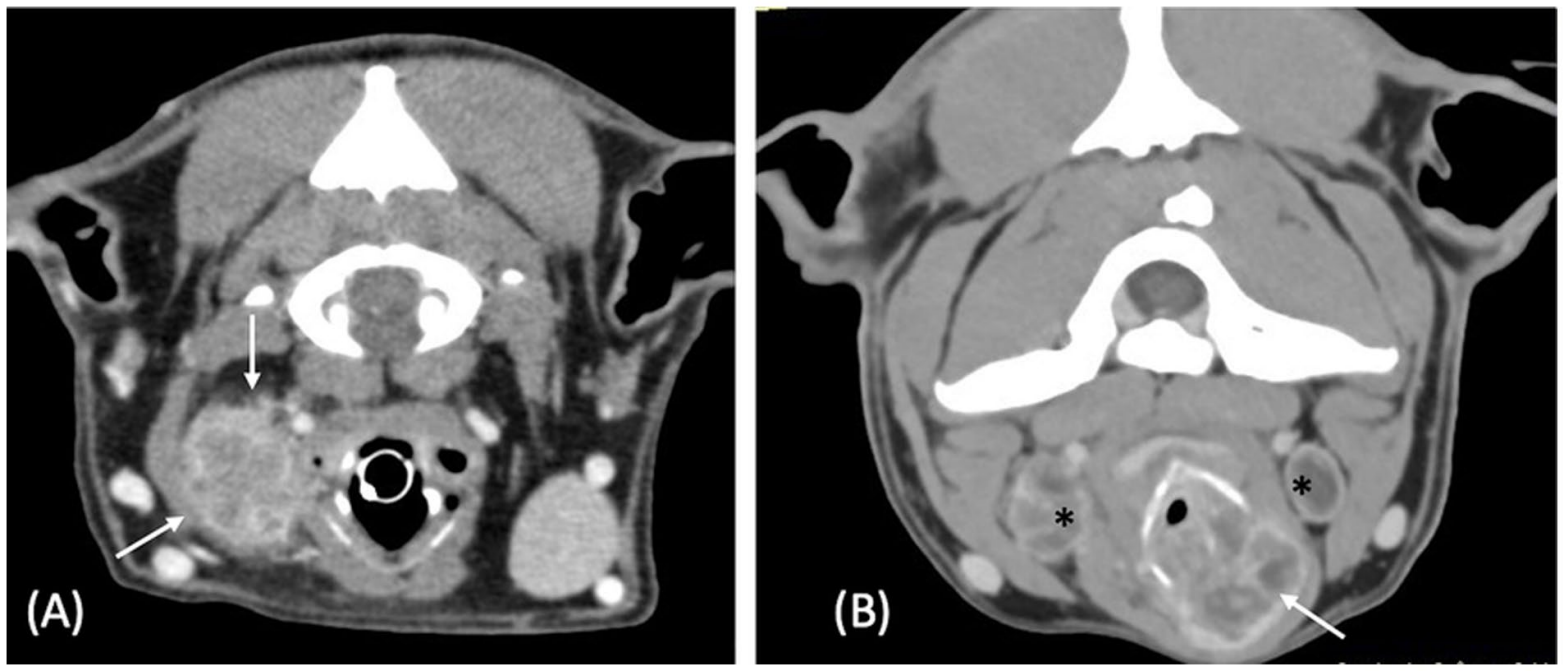
Postcontrast transverse CT images of case 1 **(A)** and case 4 **(B)**, illustrating the ovoid shape of the neoplastic laryngeal lesions (white arrows) with heterogeneous contrast enhancement. In both cases the neoplastic lesion is a carcinoma. The case 1 has a laryngeal mass with an external growth pattern and the case 4 has a laryngeal mass with mixed external and internal growth pattern. There is also moderate bilateral enlargement and heterogeneity of both medial retropharyngeal lymph nodes seen in **(B)** (*). Window width = 400 HU, window level = 40 HU.

**Figure 2:**
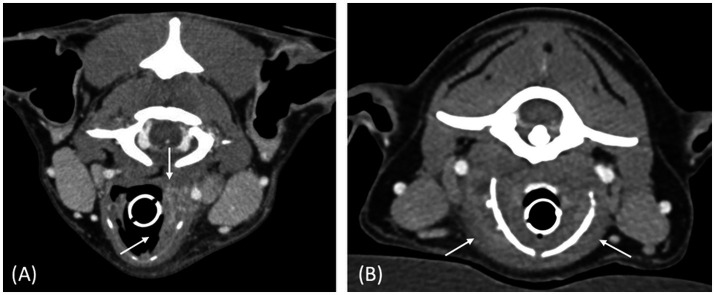
Postcontrast transverse CT images of case 3 **(A)** and case 6 **(B)**, illustrating the thickened aspect of the laryngeal lesions (white arrows). Both cases had laryngeal masses with mixed external and internal growth pattern, characterized by an increase in thickness on each side of the cartilage. Window width = 400 HU, window level = 40 HU.

All the laryngeal lesions (7/7) were described as ill-defined. All neoplastic masses (4/7) were described as ovoid-shaped (Figure 1), whereas all inflammatory lesions (3/7) were described as thickening of the larynx (Figure 2). Four lesions were unilateral, of which 3 were neoplastic and 1 inflammatory. The median length of the neoplastic lesions was 42 mm (range: 26–82 mm), the median width was 30 mm (range: 16–54 mm), and the median height was 33 mm (range: 23–68 mm). For the inflammatory lesions, the median length was 37 mm (range: 33–48 mm), the median width was 16 mm (range: 14–18 mm), and the median height was 31 mm (range: 25–38 mm).

Regarding the growth pattern of the lesions, all three inflammatory lesions exhibited a mixed internal and external growth pattern (Figure 2). Among the neoplastic cases, two showed an external growth pattern (Figure 1A), one had an internal growth pattern, and one presented a mixed internal and external growth pattern (Figure 1B).

Four lesions were associated with laryngeal cartilage destruction, of which 2 were neoplastic and 2 were inflammatory. All but one case were heterogeneous on precontrast images, and the only case with a homogeneous lesion was a neoplastic lesion. All the laryngeal lesions (7/7) were heterogeneously contrast enhancing after intravenous administration of contrast medium. Four of the lesions exhibited a cavitary component, of which 2 were neoplastic and 2 inflammatory, and 2 lesions were partially mineralized, one in each group.

All but one case had at least one enlarged regional lymph node, the only case with normal size lymph nodes was a neoplastic case. There was only one lymph node with a short-to-long axis ratio greater than 0.7, observed with a laryngeal squamous cell carcinoma. All the enlarged lymph nodes in the tumor cases exhibited heterogeneous enhancement, while in two out of the three inflammatory cases, the enhancement was homogeneous, although the nodes were still enlarged. At least one retropharyngeal lymph node was involved in all these 6 cases, followed by mandibular lymph node (3/6), parotid lymph node (2/6) and superficial cervical lymph node (2/6). When present, the adenopathy was bilateral in two of the three neoplastic cases and one out of the three inflammatory cases. Lymph node cytology was obtained from one dog with a laryngeal tumor and two dogs with an inflammatory process. In all three cases, the lymph node was identified as reactive.

Among the 7 cases, 6 underwent thoracic CT examination, all of which were unremarkable.

CT features of all the cases included in this study are summarized in Table 1 and more detailed Supporting information 1.

**Table 1:** Diagnostic imaging findings for the 4 malignant and the 3 inflammatory cases.

Diagnostic imaging findings	4 Neoplastic masses	3 Inflammatory processes
Size—Median length [range] (mm)	42 [26–82]	37 [33–48]
Size—Median width [range] (mm)	30 [16–54]	16 [14–18]
Size—Median height [range] (mm)	33 [23–68]	31 [25–38]
Margins	Ill-defined	Ill-defined
Unilateral	3/4	1/3
Cartilage destruction	2/4	2/3
Pre contrast attenuation	3/4 heterogeneous	3/3 heterogeneous
Contrast enhancement	4/4 heterogeneous	3/3 heterogeneous
Cavitation	2/4	2/3
Mineralization	1/4	1/3
Findings in thorax	Unremarkable	Unremarkable
Shape	Ovoid	Thickening
Growth pattern	1/4 internal	3/3 internal and external
	2/4 external	
	1/4 internal and external	
Regional lymphadenopathy	3/4	3/3
	2/3 bilateral	1/3 bilateral

## Discussion

The results of the present case series confirm the authors’ hypothesis that the shape of the laryngeal mass may assist in determining its nature: inflammatory processes were characterized by laryngeal thickening, whereas neoplasia presented as an ovoid-shaped mass. However, the absence of regional lymphadenopathy in one neoplastic case and its presence in all inflammatory cases contradict the initial hypothesis that lymph node size would help differentiate between the two etiologies.

In this study, only 3 masses out of the 7 where of inflammatory nature, which is not in line with a previous study in which 13 out of 15 dogs with infiltrative laryngeal disease were diagnosed with inflammatory disease (5). A selection bias could explain this difference in the proportion of inflammatory disease in comparison to neoplasia. Our cases were retrieved from referral institutions, which may indicate that inflammatory masses of the larynx may be diagnosed directly by general practitioners, via laryngoscopy, without referring the case for a CT scan of the head. Another explanation could be related to the population of dogs included in this study. Laryngeal inflammatory masses are presumed to be secondary to repetitive micro-traumas that are more frequent in brachycephalic breeds, as they usually have an increased respiratory effort and have a high prevalence of regurgitation and vomiting (5, 9, 18). In contrast to other studies, no brachycephalic dogs were included in the present study.

In people, laryngeal cancer accounts for one-third of head and neck cancers and therefore have been extensively studied (10). They are also often linked with tobacco and alcohol consumption. While alcohol is not a concern in companion animals, the effects of secondhand smoke on their health have been studied and should be kept in mind (13, 22). In people, they investigate primarily laryngeal masses with endoscopy and endoscopic biopsy and the most common indication to perform a cross-sectional imaging is the suspicion of a neoplasm (1, 4). This argues that inflammatory laryngeal masses are not frequently referred for a CT investigation in veterinary patients.

In dogs, CT investigation of laryngeal masses is performed for surgical planning. In people however the most common indication to perform a CT of the neck is to investigate about the feasibility of speech conservation therapy, which is not a concern in veterinary medicine. Moreover, it has been proven that CT has limitations for surgical management in people, as it is not sensitive enough for cartilage penetration (2, 17). This is why we should maybe reconsider the use of CT of the larynx for surgical planning, and use it more to help the radiologist prioritize a list of differential diagnoses. The CT features of human laryngeal inflammatory masses are described as massive thickening of the laryngeal mucosa, which is in line with the findings of this study (1). On the other hand, the features that are suspicious for malignancy are lesions with ill-defined margins, invasion of normal structures and osteolysis (7). In this study, all masses were characterized as ill-defined, regardless of their nature, and some cases from both neoplastic and inflammatory categories exhibited destruction of the thyroid cartilage. Therefore, criteria of malignancy used in people are not applicable to dogs.

One limitation of this study is the small cohort size, given the low incidence of laryngeal masses in dogs (21) and the fact that neck CT is not typically performed as a first-line diagnostic tool in suspected cases. Given this small cohort size, there were no other types of laryngeal lesions like for example benign masses or lymphoma of the larynx which could have different imaging features. Another limitation is the multicentric retrospective nature of this study, as imaging studies were performed with different machines, parameters, and protocols. However, these variations do not alter the shape of the laryngeal mass. Some clinical data were also partially missing, such as the results of sampling of the enlarged lymph nodes.

This study provides the first description and comparison of canine neoplastic and inflammatory laryngeal masses on CT and suggests that CT can be a valuable tool in the diagnostic process when a laryngeal mass is suspected. The findings suggest that the shape of the laryngeal mass may help determine its nature: a diffuse laryngeal thickening favors an inflammatory process, while an ovoid-shaped mass may be more suggestive of neoplasia. Neoplasia should be considered even when there is no lymphadenopathy.

## Data Availability

The original contributions presented in the study are included in the article/Supplementary material, further inquiries can be directed to the corresponding author.
