# Supplementary material for: Computed tomographic appearance of laryngeal lesions in 7 dogs
Source: Front Vet Sci. 2025 Sep 4;12:1633591. doi: 10.3389/fvets.2025.1633591 (PMC12444889; doi:10.3389/fvets.2025.1633591)
Supplement: Supplementary file 1 [file Table_1.docx]

**Supporting information 1**

NF, neutered female; NM, neutered male; F, intact female; M, intact male; H, histologically confirmed; C, cytologically confirmed; R, right; L, left; LN, lymph node; SCC, squamous cell carcinoma; Pre, preconstract CT images; PC, post contrast CT images; CrCd, cranio caudal; LM, latero medial; DV, dorso ventral
